# Deep vein thrombosis screening in pediatric orthopedic patients

**DOI:** 10.3389/fsurg.2023.1041578

**Published:** 2023-04-03

**Authors:** Saowalak Tongta, Chanika Angsnuntsukh, Tanyawat Saisongcroh, Thira Woratanarat, Yaowaret Tangsopa, Patarawan Woratanarat

**Affiliations:** ^1^ Department of Orthopedics, Faculty of Medicine Ramathibodi Hospital, Mahidol University, Bangkok, Thailand; ^2^ Department of Preventive and Social Medicine, Faculty of Medicine, Chulalongkorn University, Bangkok, Thailand

**Keywords:** Caprini score, D-dimer, Doppler ultrasound, Wells score, sensitivity

## Abstract

**Background:**

Deep vein thrombosis (DVT) is an important clinical condition that leads to subsequent morbidity and mortality in children, particularly those who involved operative procedures. The preoperative assessment for DVT in children may vary among different population risk factors and types of surgery. This study aimed to evaluate the screening methods for DVT in pediatric orthopedic patients.

**Method:**

We performed a retrospective cohort study of orthopedic patients aged <18 years at Ramathibodi Hospital, Bangkok, Thailand, from 2015 to 2019. The inclusion criteria were children scheduled for orthopedic surgery; who performed a D-dimer test, Wells score, and Caprini score; and who underwent Doppler ultrasonography for DVT screening. The exclusion criteria were incomplete data or inconclusive ultrasonographic results. Age and results of the D-dimer test, Wells score, and Caprini score were collected from all patients. The outcome assessment was ultrasound-proven DVT. The screening abilities of each test were analyzed in terms of sensitivity, specificity, positive predictive value (PPV), negative predictive value (NPV), likelihood ratio (LR) for positive and negative tests, and area under the receiver operating characteristic curve (AUC).

**Results:**

A total of 419 children were included in the study. Five (1.19%) patients were diagnosed with DVT. The mean age was 10.16 ± 4.83 years. D-dimer ≥500 ng/mL had a sensitivity of 100% (95% CI: 47.8%–100%), a specificity of 36.7% (95% CI: 32.1%–41.6%), a PPV of 1.9% (95% CI: 0.6%–4.3%), and an NPV of 100% (95% CI: 97.6%–100%). Wells score ≥3 demonstrated a sensitivity of 0% (95% CI: 0%–52.2%), a specificity of 99.3% (95% CI: 97.9%–99.9%), and an LR for a negative test of 1.00 (95% CI: 1.00–1.01). Caprini score ≥11 had a sensitivity of 0% (95% CI: 0%–52.2%) and a specificity of 99.8% (95% CI: 98.7%–100%). The parallel test included D-dimer ≥500 ng/mL, Wells score ≥3, or Caprini score ≥11 points, generating a sensitivity of 100% (95% CI: 47.8%–100%), a specificity of 36.7% (95% CI: 32.1%–41.6%), an LR for a positive test of 1.58 (95% CI: 1.47–1.70), and an AUC of 0.68 (95% CI: 0.66–0.71).

**Conclusions:**

The D-dimer test exhibited moderate ability in predicting the development of DVT among pediatric orthopedic patients requiring surgery. The Wells score and Caprini score had low performance in identifying hospitalized children at increased risk of DVT events.

## 1. Introduction

Deep vein thrombosis (DVT) is blood clot formation caused by venous stasis, vascular damage, and hypercoagulability (1). DVT occasionally occurs in children (2), with an average incidence of 0.07 per 10,000 children and 3.9–5.1 per 10,000 hospital admissions (3, 4). It is commonly found in early infancy (20%) and adolescence (50%) (5, 6). Venous thromboembolism (VTE) occurs in children undergoing orthopedic surgery at approximately 0.05% per hospital admission (3). Assessments of DVT are often made after having severe symptoms or life-threatening conditions (7). Therefore, a proper screening method would benefit early detection, early prevention, and treatment.

According to the American Society of Hematology guideline, noninvasive Doppler ultrasonography is a standard preoperative DVT screening technique (8). However, it is costly, operator-dependent, and requires experienced radiologists and surgeons. Alternate screening tools, such as the D-dimer test (sensitivity 78%–97%) (9), Wells score (sensitivity 67%) (10), and Caprini score (sensitivity 83%) (11), have been developed to identify high-risk patients. These parallel tests may detect proper cases for Doppler ultrasonography, minimize medical expenses, and save time. Moreover, these tools help reduce excess workload of ultrasound performers, especially in highly demanded institutes.

To prevent perioperative complications, preoperative DVT screening has been currently integrated into our routine clinical service system at Ramathibodi Hospital, a 1,100-bed university hospital in Bangkok, Thailand. All patients were registered in the Department of Orthopedics Database for 5 years (2015–2019). However, the screening abilities may be affected by the incidence rate of DVT, age, race, body mass index (BMI), underlying diseases, and operative procedures (3). To provide a safe and sound service system for pediatric orthopedic patients, it is necessary to find the appropriate screening methods and cutoff values to detect DVT in children who underwent orthopedic surgery. This study aimed to evaluate the DVT screening methods in pediatric orthopedic patients including the D-dimer test, Wells score, and Caprini score compared with the gold standard techinque, Doppler ultrasonography.

## 2. Methods

A retrospective cohort study was conducted by retrieving medical records and the preoperative DVT screening database from the Department of Orthopaedics, Faculty of Medicine Ramathibodi Hospital, Mahidol University, Bangkok, Thailand. All patients admitted to the orthopedic ward from 2015 to 2019 were assessed for eligibility. The inclusion criteria were children aged under 18 years; scheduled for orthopedic surgery; who performed the D-dimer test, Wells score, and Caprini score; and who underwent Doppler ultrasonography for DVT screening. The exclusion criteria were incomplete data or inconclusive ultrasonographic results. This study was approved by the Institutional Review Board (MURA2020/249).

Patient information, including age, type of surgery, DVT prophylaxis (mechanical/pharmacological methods), diagnostic tests (D-dimer test, Wells score, Caprini score), and the gold standard test (Doppler ultrasonography), was reviewed and documented. Additionally, we recorded high-risk patients with risk factors such as cancer, immobility, obesity, heart attack, congestive heart failure, infection, fracture, spinal cord injury, history of DVT, and family history of DVT. The D-dimer test, Wells score, and Caprini score were routinely evaluated for every hospitalized child before surgery. D-dimer assays were analyzed by the Clinical Laboratory Center of Ramathibodi Hospital, and cutoff level ≥500 ng/mL was set for suspected DVT. The Wells score comprises leg conditions, history of DVT/cancer, and bedridden/major surgery and is evaluated as a cumulative risk score (0–10) by orthopedic residents. Wells score ≥3 was considered a high probability of DVT. The Caprini score (0–59) was defined by baseline characteristics, surgery, history of VTE, underlying diseases, and leg conditions. Caprini score ≥11 was determined a high risk of DVT. If the D-dimer level, Wells score, or Caprini score indicated a risk of DVT, patients were brought to ultrasonographic diagnosis. Doppler ultrasound (Aplio 500; Canon Inc., Tokyo, Japan) for lower extremities was performed by well-trained radiologists or surgeons.

Statistical analysis was performed using the STATA software package, version 15.0 (Stata Corp, College Station, TX, USA). The demographic characteristics of the sample were presented as frequencies, percentages, means, and standard deviations. The association of screening was calculated using the *χ*^2^ test. The D-dimer test, Wells score, and Caprini score were compared with the gold standard, Doppler ultrasound. The screening abilities of each test were calculated as sensitivities, specificities, positive predictive values (PPVs), negative predictive values (NPVs), likelihood ratios (LRs) of positive and negative tests, odds ratios (ORs), and 95% confidence intervals (CIs). The receiver operating characteristic curve (ROC) was plotted by referring to sensitivity vs.1 − specificity. The areas under the curve (AUCs) and cutoff values were also computed. The significant *p*-value was set as <0.05. The sample size was calculated based on an alpha error of 0.05, a beta error of 0.2, a sensitivity of 99%, and an error of 10%, and the incidence of DVT was 1%. The total sample size was 404.

## 3. Results

### 3.1. Participant's demographic data

From 6,201 screened cases, 419 children met the inclusion criteria. The incidence of DVT was 1.19%. The mean age of children was 10.16 ± 4.83 years. The most common operation was pediatric orthopedic surgery (56.56%), followed by tumor surgery (12.41%) and hand surgery (10.95%). None of the participants received any mechanical or pharmacological DVT prophylaxis. According to the risk of DVT, 63.72% of children had D-dimer ≥500 ng/mL, 0.72% had a Wells score ≥3, and 0.24% had a Caprini score ≥11. The characteristics of the patients are presented in Table 1.

**Table 1:** Patient characteristics.

Variables	Number (*N* = 419)
Age (years), mean (SD)	10.16 (4.83)
Type of surgery (%)	
Foot and ankle	4 (0.95)
Hand	46 (10.95)
Pediatric	237 (56.56)
Trauma	29 (6.92)
Tumor	52 (12.41)
Spine	28 (6.68)
Sport	16 (3.82)
Hip and knee	7 (1.67)
D-dimer (%)	
≥500 ng/mL	267 (63.72)
<500 ng/mL	152 (36.28)
Wells score (%)	
≥3	3 (0.72)
<3	416 (99.28)
Caprini score (%)	
≥11	1 (0.24)
<11	418 (99.76)
Doppler ultrasound (%)	
DVT	5 (1.19)
No DVT	414 (98.81)

SD, standard deviation; DVT, deep vein thrombosis.

### 3.2. Relationship with DVT

Regarding the predictive factors for developing DVT, the DVT patients showed a significant difference in the Wells score (*p* = 0.043) compared to the non-DVT group. However, there were nonsignificant differences in age (*p* = 0.865), D-dimer level (*p* = 0.817), and Caprini score (*p* = 0.043) between both groups (Table 2).

**Table 2:** Comparisons between DVT and non-DVT groups.

Variables, mean (SD)	Doppler ultrasound		*p*-value
	DVT (*N* = 5)	Non-DVT (*N* = 414)	
Age (years)	9.80 (4.83)	10.17 (5.59)	0.865
D-dimer (ng/mL)	2,155.20 (1,113.46)	3,739.65 (15,313.72)	0.817
Wells score	0.40 (0.89)	0.07 (0.35)	0.043*
Caprini score	2.20 (1.67)	1.07 (1.66)	0.135

DVT, deep vein thrombosis; SD, standard deviation.

* Significant *p*-value <0.05.

### 3.3. Performance of screening tests

#### 3.3.1. D*-*dimer test

**Figure 1:**
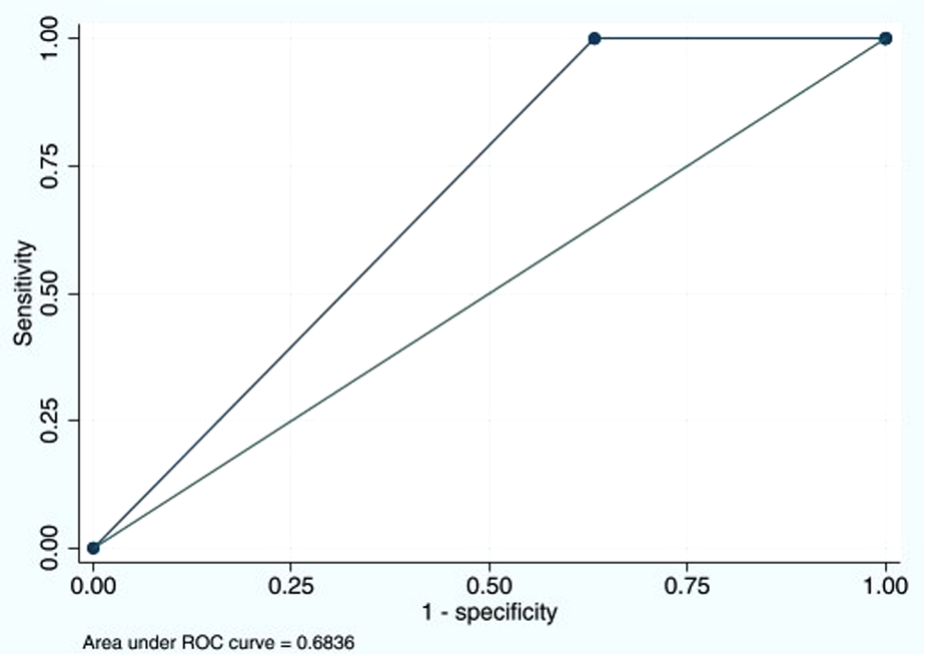
Area under the ROC curve of D-dimer cutoff ≥500 ng/mL of 0.6836. ROC, receiver operating characteristic.

The sensitivity of the D-dimer test to detect DVT was as high as 100% (95% CI: 47.8%–100%), and the specificity was 36.7% (95% CI: 32.1%–41.6%) (Table 3). The PPV was 1.9% (95% CI: 0.6%–4.3%), and the NPV was 100% (95% CI: 97.6%–100%). The LR of a positive test was 1.58 (95% CI: 1.47–1.70). The D-dimer test demonstrated good performance with an AUC of 0.68 (95% CI: 0.66–0.70) (Figure 1). Since DVT prophylaxis was not administered, the effects of anticoagulants on the D-dimer could not be assessed.

**Table 3:** Performance of screening in predicting DVT in pediatric orthopedic patients.

Tests	Doppler ultrasound (%)		Sensitivity (95% CI)	Specificity (95% CI)	PPV (95% CI)	NPV (95% CI)	Accuracy (95% CI)	LR+ (95% CI)	LR− (95% CI)	ROC area (95% CI)	OR (95% CI)
	DVT (*N* = 5)	No DVT (*N* = 414)									
D-dimer ≥500 ng/mL	5 (100)	262 (63.3)	100 (47.8–100)	36.7 (32.1–41.6)	1.9 (0.6–4.3)	100 (97.6–100)	37.5 (32.8–42.3)	1.6 (1.5–1.7)	0	0.7 (0.7–0.7)	–
Wells score ≥3	0 (0)	3 (0.7)	0 (0–52.2)	99.3 (97.9–99.9)	0 (0–70.8)	98.8 (97.2–99.6)	98.1 (96.3–99.2)	0	1.0 (1.0–1.0)	0.5 (0.5–0.5)	0 (0–125.2)
Caprini ≥11	0 (0)	1 (0.2)	0 (0–52.2)	99.8 (98.7–100)	0 (0–97.5)	98.8 (97.2–99.6)	98.5 (96.9–99.4)	0	1.0 (1.0–1.0)	0.5 (0.5–0.5)	0
D-dimer ≥500 ng/mL and Wells ≥3	0 (0)	3 (0.7)	0 (0–52.2)	99.3 (97.9–99.9)	0 (0–70.8)	98.8 (97.2–99.6)	98.1 (96.3–99.2)	0	1.0 (1.0–1.0)	0.5 (0.5–0.5)	0 (0–125.2)
D-dimer ≥500 ng/mL and Caprini ≥11	0 (0)	1 (0.2)	0 (0–52.2)	99.8 (98.7–100)	0 (0–97.5)	98.8 (97.2–99.6)	98.5 (96.9–99.4)	0	1.0 (1.0–1.0)	0.5 (0.5–0.5)	0
D-dimer ≥500 ng/mL or Wells > 3	5 (100)	262 (63.3)	100 (47.8–100)	36.7 (32.1–41.6)	1.9 (0.6–4.3)	100 (97.6–100)	37.5 (32.8–42.3)	1.6 (1.5–1.7)	0	0.7 (0.7–0.7)	–
D-dimer ≥500 ng/mL or Caprini ≥11	5 (100)	262 (63.3)	100 (47.8–100)	36.7 (32.1–41.6)	1.9 (0.6–4.3)	100 (97.6–100)	37.5 (32.8–42.3)	1.6 (1.5–1.7)	0	0.7 (0.7–0.7)	–
D-dimer ≥500 ng/mL or Wells score ≥3 or Caprini ≥11	5 (100)	262 (63.3)	100 (47.8–100)	36.7 (32.1–41.6)	1.9 (0.6–4.3)	100 (97.6–100)	37.5 (32.8–42.3)	1.6 (1.5–1.7)	0	0.7 (0.7–0.7)	–

DVT, deep vein thrombosis; PPV, positive predictive value; NPV, negative predictive value; LR+, likelihood ratio for a positive test; LR−, likelihood ratio for a negative test; ROC, receiver operating characteristic; OR, odds ratio; CI, confidence interval.

#### 3.3.2. Wells score

**Figure 2:**
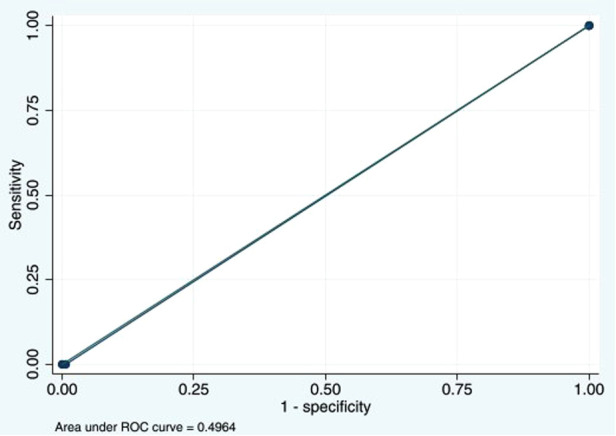
Area under the ROC curve at Wells score ≥3 cutoff point of 0.4964. ROC, receiver operating characteristic.

Wells score ≥3 could detect DVT with a sensitivity of 0% (95% CI: 0%–52.2%) and a specificity of 99.3% (95% CI: 97.9%–99.9%). The PPV was 0% (95% CI: 0%–70.8%), and the NPV was 98.8% (95% CI: 97.2%–99.6%). The LR for a negative test was 1.01 (95% CI: 1.00–1.02). The AUC was 0.49 (95% CI: 0.49–0.50) (Figure 2).

#### 3.3.3. Caprini score

**Figure 3:**
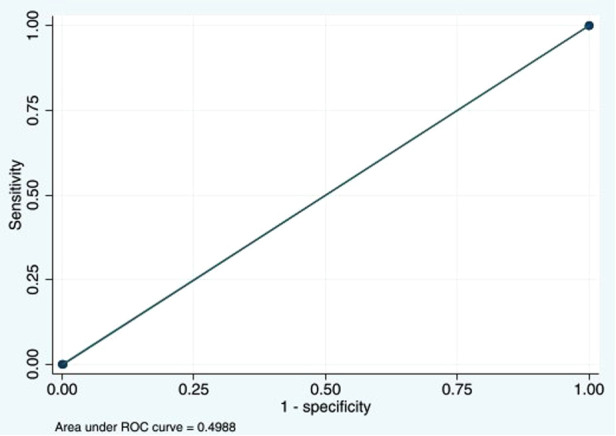
Area under the ROC curve at Caprini score ≥11 of 0.4988. ROC, receiver operating characteristic.

Caprini score ≥11 for DVT had a sensitivity of 0% (95% CI: 0%–52.2%), a specificity of 99.8% (95% CI: 98.7%–100%), a PPV of 0% (95% CI: 0%–97.5%), and an NPV of 98.8% (95% CI: 97.2%–99.6%). The LR for a negative test was 1.00 (95% CI: 1.00–1.01). The AUC was 0.49 (95% CI: 0.49–0.50) (Figure 3).

#### 3.3.4. Combination of screening tests

The combination of D-dimer ≥500 ng/mL and Wells score ≥3 provided a sensitivity of 0% (95% CI: 0%–52.2%), a specificity of 99.3% (95% CI: 97.9%–99.9%), a PPV of 0% (95% CI: 0%–70.8%), an NPV of 98.8% (95% CI: 97.2%–99.6%), and an LR for a negative test of 1.01 (95% CI: 1.00–1.02).

The combination of D-dimer ≥500 ng/mL and Caprini score ≥11 points had a sensitivity of 0% (95% CI: 0%–52.2%), a specificity of 99.8% (95% CI: 98.7%–100%), a PPV of 0% (95% CI: 0%–97.5%), an NPV of 98.8% (95% CI: 97.2%–99.6%), and an LR for a negative test of 1.00 (95% CI: 1.00–1.01) for DVT diagnosis.

For a combination of D-dimer ≥500 ng/mL and Wells score ≥3, the sensitivity was 100% (95% CI: 47.8%–100%), specificity was 36.7% (95% CI: 32.1%–41.6%), PPV was 1.9% (95% CI: 0.6%–4.3%), NPV was 100% (95% CI: 97.6%–100%), AUC was 0.68 (95% CI: 0.66–0.71), and LR for a positive test was 1.58 (95% CI: 1.47–1.70).

The combination of D-dimer ≥500 ng/mL and Caprini score ≥11 points and the parallel test including either D-dimer ≥500 ng/mL, Wells score ≥3, or Caprini score ≥11 points generated the same diagnostic abilities as the combination of D-dimer test ≥500 ng/mL and Wells score ≥3 (Table 3).

## 4. Discussion

DVT is a life-threatening condition leading to high morbidity and mortality. Identifying those who are at higher risk of developing DVT is essential to prevent fatal outcomes. This study evaluated the utility of the preoperative screening tests for DVT in children. The D-dimer test demonstrated the highest sensitivity of 100% (95% CI: 47.8%–100%) with moderate specificity of 36.7% (95% CI: 32.1%–41.6%). The Wells score, Caprini score, or combined scores provided very low sensitivity (0%–23%) with high specificity (>99%).

The development of DVT in hospitalized children is rare compared with hospitalized adults (12). Vascular endothelium in children has not deteriorated from diseases such as hypertension, diabetes, or hypercholesterolemia and other factors such as smoking or oral contraceptives (13, 14). Yen et al. estimated an incidence of 0.28% (15) among pediatric traumatic injuries and identified many risk factors to improve diagnostic sensitivity and accuracy. The incidence of DVT in our study (1.19%) was greater than the reported annual incidence (0.07–0.14 per 10,000 children) (2, 16) from other studies as well as the first report of DVT in Thai children (0.04%) in 2007 (4). The explanation for the higher incidence in this study is that we included only patients with a high risk of DVT (either D-dimer ≥500 ng/mL, Wells score ≥3, or Caprini score ≥11 points) to directly compare the utility of the screening tests used in our clinical practice and the gold standard.

**Figure 4:**
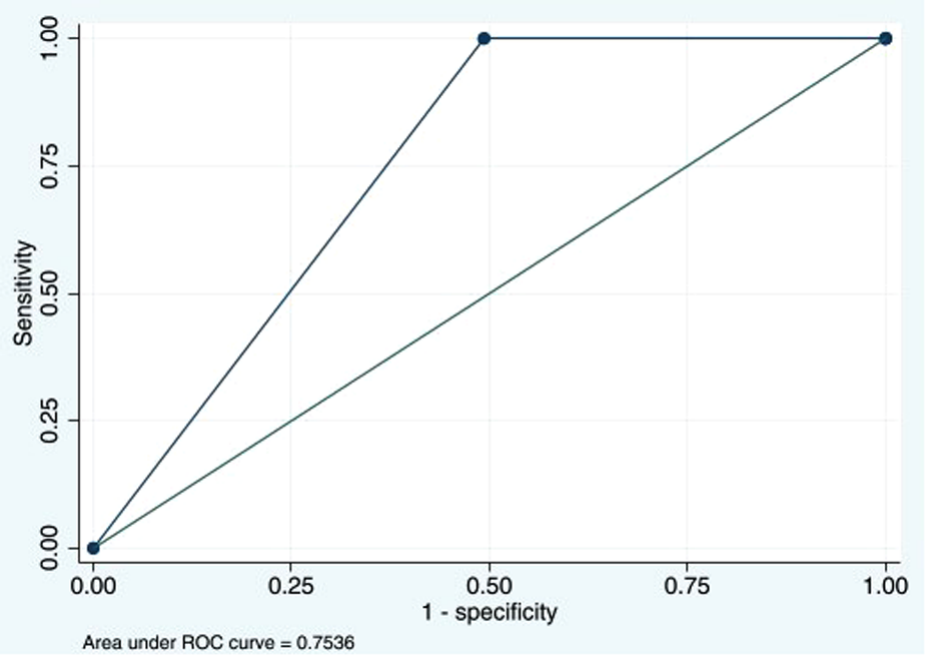
Area under the ROC curve of D-dimers with the new cutoff value at 795 ng/mL of 0.7536. ROC, receiver operating characteristic.

The D-dimer test showed high sensitivity but relatively intermediate specificity at cutoff ≥500 ng/mL and AUC at 0.7. Our test performance was similar to that in Kanis et al. (17) and other previous studies (18, 19) that demonstrated high sensitivity of the D-dimer test for DVT. Additionally, a meta-analysis in adults by Stein et al. reported that the sensitivity and specificity of the D-dimer test at cutoff >500 ng/mL ranged from 78% to 97% and 42% to 70%, respectively (9). Nevertheless, the diagnostic ability may relate to patients’ characteristics, age, underlying conditions, D-dimer assays, and the chosen cutoff values (19). The D-dimer in our study had 100% sensitivity and 100% NPV, but the specificity and PPV for DVT were quite low. From our thorough analysis, we have identified new cutoff values at 795 ng/mL to improve the diagnostic performance. This level provided a sensitivity of 100% (95% CI: 47.8%–100%), a specificity of 50.7% (95% CI: 45.8%–55.6%), and an AUC of 0.8 (95% CI: 0.7%–0.8%), as shown in Figure 4. The review of studies in children with the D-dimer test for DVT is shown in Table 4.

**Table 4:** Reviews of studies with D-dimer tests for deep vein thrombosis.

Author	Year	No. patients	Age (year)	Cutoff (ng/mL)	Sensitivity (%)	Specificity (%)
Strouse et al. (19)	2009	33	0–21	1,500	96	43
Kanis et al. (17)	2018	526	5–17	500	89	56
This study	2022	419	0–18	500	100	37
This study	2022	419	0–18	795	100	51

Although the Wells score was used to determine the probability of developing DVT in patients, its screening efficacy, specifically in children, has not been reported. Our study presented the largest sample of children and found that Wells score ≥3 had a sensitivity of 0% (95% CI: 0–52.2) and a specificity of 99.3% (95% CI: 97.9–99.9). Their results showed high specificity without sensitivity for DVT. The Wells score performed poor discrimination (AUC, 0.49) to spot the risk of DVT in pediatric orthopedic patients requiring surgery. Silveira et al. reported a low discriminatory rate of the Wells score for inpatients (AUC, 0.56) (20). Nevertheless, a high Wells score is associated with an increased probability of DVT (10, 21). Our results suggest that the Wells score risk stratification might not be applicable for pediatric orthopedic patients due to different risk factors of DVT between children and adults (14).

Patients with higher Caprini scores were correlated with an increased likelihood of VTE events. The Caprini score of 11 can identify the high-risk subgroup among patients requiring surgery. From our study, Caprini score ≥11 hardly rules out the possibility of DVT with a sensitivity of 23.9% (95% CI: 17.9%–30.8%), but it effectively rules in with a specificity of 99.1% (95% CI: 98.8%–99.3%). Contrarily, Luksameearunothai et al. reported the sensitivity and specificity of Caprini score ≥12 among the elderly of 93% and 35%, respectively (22). Hachey et al. also found that the Caprini score produced a sensitivity of 83.3% and a specificity of 60.5% among patients who underwent lung cancer resection (11). We suggest that the preoperative Caprini score may not be used to identify DVT risk among children who underwent orthopedic surgery.

Based on the ROC analysis, the AUC of the D-dimer test has a high utility in the diagnosis of DVT. We tried to combine the scores in a serial or parallel test. The combination of the D-dimer and Wells score resulted in 0% sensitivity, 99.8% specificity, 0% PPV, and 98.8% NPV. For the parallel test, they had the same diagnostic values as those of the D-dimer test, i.e., 100% sensitivity, 36.7% specificity, 1.9% PPV, and 100% NPV, for DVT diagnosis. Therefore, combining other tests with the D-dimer test did not provide better diagnostic abilities.

Surgical procedure is one of the major risk factors, especially for those with longer operative times and longer hospital stays (23). In addition, underlying diseases or types of surgery might influence the incidence of DVT. Of five DVT cases in our study, three had undergone tumor surgery, one pediatric surgery, and one spine surgery. Our results demonstrate that 60% of DVT cases underwent tumor surgery. This reflects that the malignancy may be strongly associated with DVT (13, 24, 25). This association could be explained by several mechanisms. First, the tumor may put pressure on the vein, resulting in blood cell stasis and endothelial damage in the blood vessels. Others may include cancer-associated thrombosis due to the direct effect of the cancer cells on stimulating blood clot formation, secreting platelets and thrombin, and increasing the risk of developing deep vein thrombosis (26, 27). From our study, the average D-dimer level of orthopedic tumor cases was significantly lower than that of non-tumor patients (*p* = 0.042). However, there was no significant difference in the high D-dimer level cutoff of 500 ng/mL between the tumor (55.8%) and non-tumor (64.9%) groups with *p*-value = 0.202. The explanation is that conditions other than tumors, such as trauma, infection, inflammation, cardiac, and renal diseases, also contribute to D-dimer elevation, possibly by fibrin clot activation (18).

The strengths of this study are that we conducted it on the registered database with a standardized data collection form and a well-established system with integrated DVT screening tests in routine clinical services. There are still some limitations as our design is a retrospective study. First, database review may have unknown or unreported history leading to underestimating certain DVT risks. Second, the radiologists and surgeons who performed the Doppler ultrasound may be aware of the screening test results. Third, the incidence of DVT may be overestimated due to the inclusion of the cases with at least one screening test positive as a prerequisite for Doppler ultrasonography. However, this represented the real situation for DVT screening in our context. Finally, diagnostic abilities showed a wide and imprecise 95% CI of odds ratio (Table 4), reflecting the small sample size of the study. Since the incidence of DVT is very low in children, recruitment of more study populations would be of value for future research.

## 5. Conclusion

The D-dimer test produces moderate performance in predicting the development of DVT. However, the Wells score and Caprini score had poor performance. Even though clinical assessment tools are practical and useful for categorizing risks of DVT, we recommend the D-dimer test for preoperative DVT screening in pediatric orthopedic patients. Based on this study, a D-dimer cutoff value of 795 ng/mL is the most appropriate for DVT screening among pediatric orthopedic patients requiring surgery. A further study is needed to externally validate the new D-dimer cutoff value.

## Data Availability

The original contributions presented in the study are included in the article/Supplementary Material; further inquiries can be directed to the corresponding author.
